# Reimagining policy implementation science in a global context: a theoretical discussion

**DOI:** 10.3389/frhs.2024.1292688

**Published:** 2024-09-20

**Authors:** Kellie List, Peter Agamile, Didier Yélognissè Alia, Peter Cherutich, Kristen Danforth, John Kinuthia, Arianna Rubin Means, Irene Mukui, Nancy Ngumbau, Yanfang Su, Anjuli Dawn Wagner, Bryan J. Weiner, Sarah Masyuko

**Affiliations:** ^1^ Department of Global Health, University of Washington, Seattle, WA, United States; ^2^ Daniel J. Evans School of Public Policy & Governance, University of Washington, Seattle, WA, United States; ^3^ Kenya Ministry of Health, Nairobi, Kenya; ^4^ Medical Research & Programs Department, Kenyatta National Hospital, Nairobi, Kenya

**Keywords:** policy, implementation science, global context, policy cycle, global health

## Introduction

Policy implementation science (IS) is defined here as generating knowledge and deploying implementation strategies to effectively adopt and integrate evidence-based interventions (EBIs) into policy designs and improve policy implementation and effectiveness (1–3). Most existing policy IS scholarly works originate from the Global North (United States, Canada, and Western Europe) and describe or evaluate strategies to increase the uptake of EBIs (4). The existing Global North-generated frameworks focus less on the critical resources needed to formulate and implement a policy in diverse settings. Current approaches to policy IS lack sufficient contextual nuance to be applicable to a broader global population and limit the potential impact of policy IS in low-resource settings. Globally, there are differences in the emphasis of universal healthcare (UHC) vs. individualized healthcare, the value of affordability vs. gross domestic product (GDP) or per capita spending on health, and access to health insurance and availability of primary care vs. specialized care. Many countries of the Global South have mixed healthcare systems, comprising both public and private sectors, with the majority of the population relying on public health services. The governance of health systems is often a mix of centralized and decentralized models with shared responsibilities between the national government and subnational systems either as states, regions, or counties. These subnational levels have some degree of autonomy to make localized policies. Health systems in many Global South countries are characterized by considerable resource scarcity in funding, workforce, medicines and medical technologies, and equipment (5). However, there are still some variations within the Global South. For example, Colombia, a middle-income country with a mixed public–private healthcare system, has high insurance coverage. Nearly all Colombians (99%) are enrolled in a collection of state and private insurance companies; they receive annual allocations depending on their number of enrollees from a centrally managed government pool fund, giving the national government substantial oversight authority and responsibility in regulating insurers (6). Similarly, variations exist in the Global North systems. For example, the United States has a privatized system, whereas the United Kingdom has a system more focused on the public sector. These governance and sociopolitical differences—especially in health system decentralization, resources, and prioritization of outcomes (7–9), as well as the history of colonization and resulting international donor power—mean that the direct application of a Global North-generated policy IS framework may be less effective in a global context.

Utilizing the policy cycle heuristic of agenda setting, policy formulation, and policy implementation and evaluation (10), we highlight domains within policy IS frameworks that could be strengthened with traditional IS frameworks and political science scholarship to be more applicable within heterogeneous settings. Numerous IS frameworks have been robustly informed by scholarship in the Global South, which reflect many types of power differentials and emphasize the criticality of stakeholder input and reciprocal collaboration (11, 12). However, these traditional IS frameworks often lack explicit considerations for agenda setting, policy formulation, and policy implementation (13, 14). Although political science scholarship has focused extensively on the policy cycle, particularly agenda setting, with dozens of relevant theories, few (15) have been developed explicitly outside of a Global North setting. The unique case of international donors in health policy processes is less reflected in these theories. The policy IS frameworks developed to bridge this gap between policy and IS have opportunities for refinement to adequately reflect the policy processes (16) and the evolving role of donors as unique stakeholders (17). Therefore, more work is needed to expand policy IS frameworks by incorporating more traditional IS and political science theories to make global policy implementation scientists better stewards in global policy processes. By outlining potential concepts for future scholarship, we hope to advance the use of policy IS globally to address global goals such as reducing inequities, moving forward to a more just world, and decolonizing global health.

### Agenda setting

Policy IS frameworks—including those by Crable et al. and Bullock et al. (16, 17)—often assume, either implicitly or explicitly, that the role of the researcher (or potentially the funder) is to persuade the policymaker (18, 19). This results in a focus on disseminating research findings to policymakers, with less attention on other relevant stakeholders in the process. The unidirectional assumption may be particularly inappropriate in global health settings where the policy process can be either more or less centralized, include diverse sets of stakeholders, and where the history of colonization and the outsized power of international donors are acutely likely to influence the development of national and subnational health agendas. In contrast, existing political science theories on agenda setting describe policy and policy implementation as non-linear processes. The HIV guideline and policy development process in Kenya is a good example of reciprocal relationships between researchers, donors, policymakers, community members, and other stakeholders. This multiorganizational and multidisciplinary process involves multiple actors convened as a technical working group or task force, a deep review of existing literature on topics presented by researchers, a review of lessons learned from demonstration projects, mapping of available resources at subnational levels, and identifying additional questions to be subsequently included in the research agenda. One notable example is the public sector adoption and scale-up of oral pre-exposure prophylaxis (PrEP) in Kenya from 2016 onward. Local scientists were involved in generating the evidence that led to the global adoption of PrEP, and large-scale demonstrations were conducted in the country. This contributed to a high level of trust and goodwill toward the evidence that supported the introduction of PrEP, which played a part in facilitating the acceptance and scaling of the PrEP program (20).

**Figure 1:**
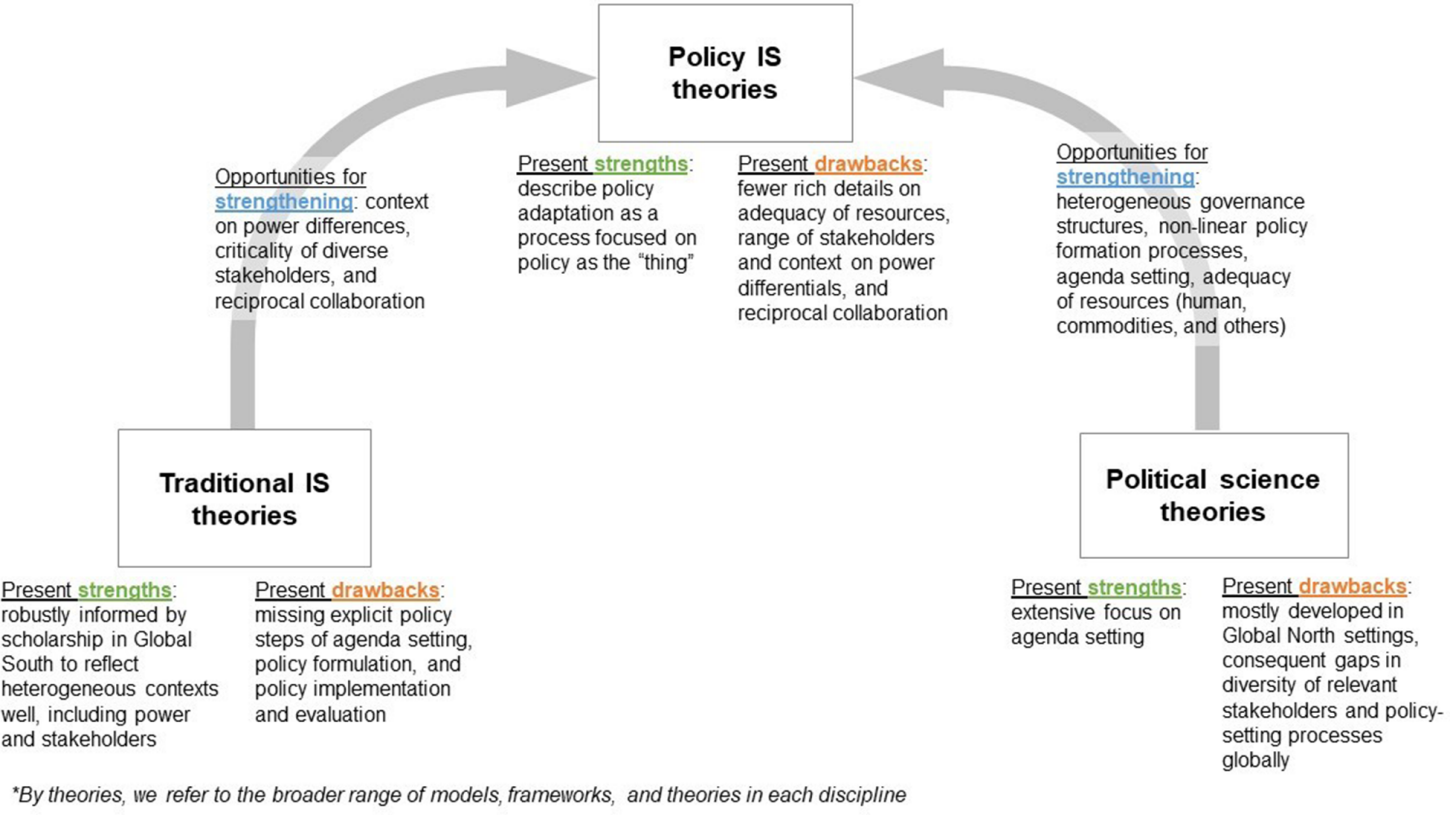
Strengths and opportunities for improvements in theories from the related disciplines of political science research, traditional implementation science theories, and policy implementation science. The arrows demonstrate how strengths from one discipline can help improve existing policy implementation science theories.

In the absence of agenda-setting-oriented IS frameworks, multiple researchers have used Kingdon's “multiple streams” theory to explore health priorities in the Global South. This theory centers on the opening of a “policy window” when the appropriate problem, policy, and political streams align, which allows for a policy change (21). This framing is easy to comprehend when the power structures for the actors within those streams ebb and flow relatively equally over time. However, in practice, the balance of power among stakeholders (both internally and externally) differs in countries in the Global South due to governance structures, health system infrastructure, varied levels of resources, sources of funding, and histories of colonization (22). The power and roles of individual actors and external stakeholders (including bilateral and multilateral institutions, philanthropic actors, normative guidance institutions (e.g., World Health Organization), and communities vary. The involvement of these multilayered actors starts early and spans from testing evidence-based interventions to their implementation and scale-up. External actors may have their own agendas and be less susceptible to influence by local actors, echoing the dynamics of colonialism and international development. For example, while local actors have advocated for chronic diseases to feature prominently in the health policy agenda, external actors continue to prioritize and fund infectious disease programs (23). Often, this decision-making power is linked to resource availability to fund programs. To more effectively advance the locally led health agenda-setting process, policy IS frameworks and practitioners need a better understanding of how to navigate entrenched power structures. To address this, policy IS theories and frameworks should be expanded to include reciprocal relationships between policy actors and account for varying governance structures (Figure 1).

### Policy formulation

Across policy formulation, there is an acknowledgment that policies need to be adapted when transferred to different country contexts. However, the process for doing this has not received the same intensity of study as the adaptation and translation of evidence-based interventions, which is common in IS frameworks (24, 25). Typically, policy adaptation is conceptualized as a technocratic process—updating targets and implementation instructions based on country-specific data.

Current approaches to a benefits package design, adapted heavily from experiences in the United Kingdom (NICE), Thailand, and other European health systems, assume highly centralized, data-intensive, nominally apolitical governance structures for health, with policy formulation decision-making centered within a central figure or office, disseminating guidance to other local entities. This approach has proven insufficient for decentralized systems, where significant autonomy is reserved for local or subnational levels, with broader guidelines established by a central figure or office. While countries with differing governance structures have created and implemented UHC policies, implementation in the Global South has lagged due to a lack of financial and human resources (26, 27), which remain key contextual factors that need to be considered when adapting policies for heterogeneous settings.

We present two case studies that highlight the need to adapt a general policy to fit different governance structures for health and various financial and human resource contexts when developing a universal health coverage essential benefits package. In the most recent revision to its Essential Package of Health Services for the 12th Five-Year Plan, Pakistan identified efficiencies and alignment with evidence-based recommendations by using the normative package proposed in the 3rd edition of Disease Control Priorities (DCP3) as a framework. Through a year-long collaborative process led by the Ministry of National Health Services, Regulations & Coordination, the initial policies proposed in the DCP3 package were narrowed and adapted into a set targeted to the needs of the Pakistani population and aligned with the health system structure that centers on a community-focused delivery model (28, 29). Rwanda, on the other hand, approached the achievement of universal health coverage by strengthening its primary healthcare infrastructure and implementing a community-based health insurance program (*Mutuelle de santé*) (30). The program provides health insurance coverage to more than 90% of its population, resulting in improved health outcomes. This success is attributed to strong leadership and community partnerships, as evidenced by the increasing government spending on health, exceeding the regional average, and the role of the community in its implementation (31–33). The approaches taken by these two countries in developing UHC demonstrate the necessary adaption of this general policy to fit the local context.

Recent policy IS frameworks uniquely describe policy as the focus of adaptation. In the original Exploration, Preparation, Implementation, and Sustainment (EPIS) framework, policy was conceptualized as a contextual “bridging factor” that connects outer and inner contexts (34). In the policy-optimized version of the EPIS framework, policy is conceptualized as the “thing” to be tailored (16) (Figure 1). These frameworks also present an opportunity for expansion to reflect contextual factors such as governance structures and the role and power of external actors—areas better articulated by more traditional implementation science frameworks informed by Global South scholarship (11, 12) (Figure 1).

### Policy implementation and evaluation

Arguably, most theoretical and empirical work in policy IS to date has been conducted on the latter phases of the policy cycle. While the integration of implementation science concepts into policy implementation research has made great strides since Nilsen et al. (35) first lamented their incompatibility, the language used in IS research on policy still reflects assumptions of the political economy of the Global North. Current frameworks stress the role of advocacy coalitions, organizational networks and capacity, and the influence of types of policy levers but gloss over the impact of extreme resource scarcity on implementation outcomes. Policy implementation in any context, particularly in health, is affected by substantial heterogeneity in capacity (workforce, resources, commodities) at national, subnational, and facility levels, and in the Global South, this heterogeneity is amplified not only by an acute lack of funding compared to need but also by the stark lack of fungibility of resources (36). Bullock et al. (17), in their seminal work on policy implementation theory, note that the overrepresentation of studies from the United States limits the field's consideration of other resource allocation models. However, their final determinants framework does not include resource adequacy in the model (Figure 1). Finally, while there is little published scholarship on whether implementation outcomes should differ between resource-rich and resource-constrained settings, this has been a topic at recent dissemination and implementation conferences (37).

As with agenda setting and formulation, in the Global South, external funders and program implementers exert unique influence on policy implementation outcomes. Development agencies and international non-governmental organizations (NGOs) acting in the Global South limit the extent to which governments can fully manage the policy implementation process. This may be driven by government expenditures on health. For example, in 2021, health expenditure in high-income countries stood at 13.13% of their GDP, while the corresponding figure was 5.25% in low-income countries (38). Country-level comparisons reveal even more stark differences in health expenditures. For example, the United States spent 17.36% of its GDP on health, while two of Africa's most populous countries, Ethiopia and Nigeria, only spent 3.21% and 4.08%, respectively (5). Countries with low health expenditures are dependent on financial assistance from high-income countries, and this commonly comes with their set of policy priorities. Paina et al. (39), Qiu et al. (40), and Carbaugh (41) documented how the 2015 President's Emergency Plan for AIDS Relief (PEPFAR) directive to their country missions to transition HIV/AIDS funding away from low-burden areas to increase efficiencies in programming had an undeniable influence on HIV policy implementation in the respective countries. Government and external funders play a significant role especially in geographical prioritization and transition efforts requiring government financing. NGOs play an outsized role in policy implementation and evaluation, and these NGOs are accountable to governments in complex and varying ways, directly reflecting the history and aftermath of colonial rule. Solutions to these challenges will be multifactorial and relate to governance and power shifting, similar to how decolonizing global health involves critically revising power and governance relationships and structures.

With regard to areas for further improvement in policy implementation and evaluation, policy IS frameworks could be refined to reflect the role and power of external actors using insights from more traditional implementation science frameworks (Figure 1).

## Discussion

It is clear from our discussion that we need to continue refining policy implementation science frameworks to fully embrace a global perspective addressing differences in governance, resources, and stakeholder relationships. This presents an opportunity to reduce inequities and prioritize decolonizing global health. By expanding policy IS frameworks through the incorporation of more traditional IS and political science theories and advancing an intersectionality approach that recognizes complex relationships and the impact of power dynamics on policymaking in a global setting, countries can better adapt policies to their local socio-cultural, economic, and political contexts. This should occur not only at the policy implementation and evaluation stages but also at all upstream stages in the policy cycle, such as agenda setting and policy formulation. It will be essential to move beyond this theoretical work and toward empirical research to make this agenda a reality. We propose that the future roadmap for this research includes engaging with diverse stakeholders using formative and consensus-driving methodologies to integrate policy frameworks, implementation science frameworks, and policy IS frameworks with a global health lens. We then propose a review of this integrated work by individuals in diverse contexts, applying these integrated frameworks to previous case studies to determine whether they resonate more strongly than the unadapted versions. Finally, we would propose the prospective application of such frameworks to policy IS work in global contexts.

Ultimately, using policy implementation science to promote the uptake and adoption of evidence-based policymaking presents a unique opportunity available to countries and needs to be broadened to ensure effectiveness in the Global South. It will be essential to move beyond this theoretical work toward empirical research to make this agenda a reality. This will require more interdisciplinary work, bringing together experts in implementation science, public policy, social science, and health equity, among others, to further advance the global application of policy implementation science.
